# Antifungal Strategy in Patients with Invasive Fungal Disease Associated with Hematological Malignancies Based on Risk Stratification

**DOI:** 10.1155/2022/1743596

**Published:** 2022-04-07

**Authors:** Lijin Chen, Luting Luo, Yanxin Chen, Yinzhou Wang, Jing Li, Xiaoyun Zheng, Ting Yang, Jianda Hu

**Affiliations:** ^1^Department of Hematology, Fujian Institute of Hematology, Fujian Provincial Key Laboratory of Hematology, Fujian Medical University Union Hospital, Fuzhou 350001, Fujian, China; ^2^Department of Oncology, Affiliated Quanzhou First Hospital of Fujian Medical University, Quanzhou 362000, Fujian, China; ^3^The Second Hospital of Sanming, Sanming 366000, Fujian, China

## Abstract

Patients with hematological malignancies (HM) often develop the invasive fungal disease (IFD), causing important morbidity/mortality. While treatment guidelines are available, risk stratification models for optimizing antifungal therapy strategies are few. Clinical records from 458 HM patients with IFD were retrospectively analyzed. Following Chinese treatment guidelines, patients received empirical (*n* = 239) or diagnostic-driven therapy (*n* = 219). The effectiveness rate was 87.9% for the empirical and 81.7% for the diagnostic-driven therapy groups (*P* ≥ 0.05). The incidence of adverse reactions was 18.4% and 16.9%, respectively (*P* ≥ 0.05). All risk factors of IFD in HM patients were estimated in the univariate analyses and multivariate analyses by the chi-square test and logistic regression model. Duration ≥14 days (OR = 18.340, *P*=0.011), relapsed/refractory disease (OR = 11.670, *P*=0.005), IFD history (OR = 5.270, *P*=0.021), and diabetes (OR = 3.120, *P*=0.035) were significantly associated with IFD in the multivariate analysis. Patients with more than 3 of these factors have a significant difference in effective rates between the empirical (85.7%) and diagnostic-driven (41.6%) therapy (*P*=0.008). Empirical and diagnostic-driven therapy effective rates were 80.6% and 70.9% in the patients with two risk factors (*P* > 0.05) and 85.1% and 85.4% in the patients with one risk factor (*P* > 0.05). Thus, there was no significant difference in effectiveness in patients with one or two risk factors. The abovementioned risk stratification can guide clinical antifungal therapy. The patients with 3 or more risk factors benefit from empirical therapy.

## 1. Background

Invasive fungal disease (IFD) refers to the pathophysiological processes and changes that result from fungi invading, growing, and reproducing in human tissues and blood, leading to tissue damage, organ dysfunction, and inflammation. IFD is uncommon in the general population, but is often observed in patients with immunodeficiencies and is an important cause of morbidity and mortality in patients with hematological malignancies (HMs). The incidence of IFD in patients with HMs has been rising in recent years due to the extensive use of chemotherapy, radiotherapy, broad-spectrum antibiotics, glucocorticoids, immunosuppressive agents, central venous catheterization, and hematopoietic stem cell transplantation (HSCT). Diagnosis of IFD is based on the positive culture in samples obtained aseptically or on histo/cytopathologic examination of tissue biopsies. *Candida* and *Aspergillus* strains are the main invasive fungi and molds in these patients. Nevertheless, early diagnosis of IFD is problematic because of the lack of specific clinical features and imaging testing, the poor ability to detect pathogens, and the high risk associated with tests such as lung biopsies. Empirical therapy with antifungal drugs such as voriconazole or amphotericin B has been used as initial treatment in neutropenic patients with ineffective antibacterial therapy and recurrent fever. However, empirical therapy has side effects, can induce drug resistance [1], and is expensive. Furthermore, with the continuous progress of imaging and laboratory testing, diagnostic-driven therapy has started to emerge. A meta-analysis of comparison between empirical therapy and diagnostic-driven therapy showed that diagnostic-driven therapy can effectively reduce the mortality rate related to IFD without increasing the use of antifungal drugs [2]. To date, the choice between empirical therapy and diagnostic-driven therapy is still controversial but should be made taking into consideration the risk of infection, the patient's drug tolerance, and economic conditions.

Risk assessment of infection in patients with HMs is useful to identify high-risk patients who might benefit from early intervention. In recent years, risk stratification models for IFD have been put forward by many scholars. Risk factors currently reported to be associated with IFD in HMs include neutropenia, relapsed/refractory disease, acute leukemia, complications (pulmonary dysfunction, diabetes, hypoalbuminemia, etc.), history of fungal infection, and long-term use of glucocorticoids.

In this study, the clinical characteristics from 458 patients with HMs and IFD followed in the Hematology Department of Fujian Medical University Union Hospital were retrospectively extracted and analyzed. Diagnostic accuracy rate, effectiveness rate, and adverse reaction rate between empirical and diagnostic-driven therapies were compared. Based on existing domestic and foreign guidelines and the literature, recognized risk factors were selected to establish a risk stratification model for IFD, and the validity of the model on the choice of treatment strategy was tested.

### 1.1. Patients and Methods

#### 1.1.1. Retrospective Cohort

Charts from patients with HM associated with IFD and hospitalized between January 2016 to June 2018 were retrospectively analyzed to extract clinical and laboratory data.

#### 1.1.2. Diagnostic Criteria

IFD diagnosis was made according to the Chinese guideline [3] (see Table 1 for details).

HM diagnosis was made according to Zhang Zhinan's criteria (3rd edition) [4]. Malignant HMs included acute myeloid leukemia (AML), acute lymphoid leukemia (ALL), hybrid acute leukemia (HAL), myelodysplastic syndrome (MDS), multiple myeloma (MM), malignant lymphoma (Hodgkin lymphoma/non-Hodgkin lymphoma, HL/NHL), chronic myeloid leukemia (CML), and chronic lymphocytic leukemia (CLL). All patients were subjected to cytomorphology, histological chemistry and biopsy of bone marrow, subtyping by flow cytometry, detection of the fusion gene, and chromosome examination to confirm the diagnosis of HMs. All patients with HMs initially received the recommended first-line chemotherapy. Patients who relapsed or had refractory disease received second-line treatment.

#### 1.1.3. IFD Treatment Groups

Patients were treated according to the guidelines from the Chinese Invasive Fungal Infection Working Group [3] for the diagnosis and treatment of IFD in patients with HM and cancers (5th edition) and received either empirical (*n* = 239) or diagnostic-driven therapy (*n* = 219).

For the empirical group, the antifungal treatment was initiated when broad-spectrum antibiotics given for 4–7 days were ineffective and fever persisted or when fever reoccurred after 4 or 7 days of antibiotics and there was no imaging or microbiological evidence of IFD [3]. The antifungal therapy was continued until the patient's temperature returned to normal or clinical symptoms improved.

For the diagnostic-driven treatment group, antifungal therapy was initiated if any of the following conditions occurred, e.g., imaging examination suggesting pneumonia, acute sinusitis, stage III mucositis, or most importantly, septic shock, IFD-related skin damage, central nervous system symptoms with unknown etiology, liver or spleen abscess, severe diarrhea, colonization by *Aspergillus*, or positive (1, 3)-b-D-glucan (G test) and/or galactomannan tests (GM test). The antifungal therapy was continued until the patient's imaging changes disappeared or microbiological evidence became negative [3].

#### 1.1.4. Treatment Outcomes

Antifungal therapy was considered effective when patients recovered from fever during neutrophil deficiency and were still alive and without new fungal infection 7 days after the start of antifungal treatment. At the end of treatment, clinical symptoms had improved or were completely relieved and imaging and laboratory tests improved or became negative [3]. Antifungal therapy was not stopped because of side effects.

Antifungal therapy was considered ineffective when patients experienced aggravation or no improvement of the clinical symptoms after 7 days of drug use, and imaging and microbiological testing did not improve or suggest progress. [3].

Death was recorded as directly or indirectly related to IFD [3].

#### 1.1.5. Risk Factors of IFD

The risk factors previously reported in various guidelines [3, 5, 6] and the literature [7–11] included primary disease, neutropenia duration, disease status, IFD history, complications, use of glucocorticoids, high-dose chemotherapy, hypoproteinemia, central venous catheterization, male, and age. Based on previous guidelines and literature, this study included the following risk factors: primary disease, disease status, neutropenia duration, use of glucocorticoids, IFD history, diabetes, pulmonary disfunction, hypoproteinemia, central venous catheterization, male, and age.

#### 1.1.6. Methods

**Figure 1:**
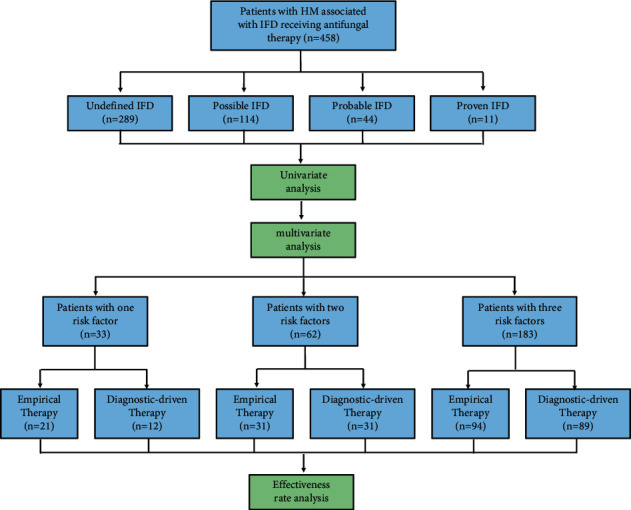
Flowchart illustrating the population and design of this study.

Patients' sex, age, primary disease, disease status, chemotherapy, neutropenia duration, history of IFD, use of glucocorticoids, complications, etc. were recorded. Effectiveness rate and adverse reaction rate in the empirical and diagnostic-driven therapy groups were compared. The risk factors of IFD in 458 patients with HM were identified by univariate and multivariate analyses. The effectiveness rate of antifungal treatment was evaluated in patients with a different number of risk factors. The effectiveness rate is defined as the percentage of patients with effective treatment, as outlined above [12] (Figure 1).

The data were analyzed with the SPSS 24.0 software. Data with normal distribution were expressed as the mean ± standard deviation, and Student's *t*-test was used for comparison between groups. Nonnormal distribution data were represented by median *M* (P25, P75), and the nonparametric rank-sum test was used for comparison between groups. Enumeration data were expressed as rate or ratio. The chi-square test was used for univariate analyses. The logistic regression model was used for multivariate analyses. Significance was set at *p* ≤ 0.05.

## 2. Results

### 2.1. Patients' Characteristics

A total of 458 HM cases were included in the study, including 285 males and 173 females, with a median age of 53 (39, 62) and neutropenia duration with the treatment of 11.4 ± 10.5 days. Primary diseases included 233 AML (50.9%), 61 ALL (13.3%), 2 HAL (0.4%), 29 MDS (6.3%), 32 MM (7.0%), 91 HL/NHL (19.9%), 3 CML (0.7%), and 7 CLL (1.5%). Twelve patients underwent HSCT. Among the 458 patients, 210 (45.8%) were newly diagnosed cases, 43 (9.4%) were in complete response (CR), 97 (21.2%) were in relapsed/refractory/no-remission, and 108 (23.6%) were in partial response (PR)/stable disease (SD). There were 239 cases in the empirical treatment group and 219 cases in the diagnostic-driven treatment group. There was no significant difference in sex, age, primary disease, disease status, chemotherapy, IFD history, diabetes history, or glucocorticoid use between the two groups (*p* ≥ 0.5). The average neutropenia duration was 12.38 days in the empirical treatment group and 10.35 days in the diagnostic-driven group, and the difference between the two groups was significant (see Table 2 for details).

### 2.2. Pathogens

A total of 187 clinical isolates were positive for fungi/molds. The source of specimens was sputum (*n* = 75; 40.1%), feces (*n* = 68; 36.4%), oral cavity (*n* = 20; 10.7%), pharynx (*n* = 10; 5.3%), blood (*n* = 8, 4.3%), perianal (*n* = 3; 1.6%), and midstream urine (*n* = 3, 1.6%). *Candida* strains were most often detected (181 cases; 96.8%), with only 5 cases of *Aspergillus* (2.7%) and 1 of *Fusarium* (0.5%) (see Table 3 for details).

### 2.3. Infection Site Distribution

There were 458 patients with 615 sites infection, 323 (70.5%) patients with one site infection (313 in the lung, 4 in the intestine tract, 4 in the oral cavity, 1 in bloodstream, and 1 in the urinary tract), and 109 (23.8%) patients with two sites infection (60 in the lung and intestines, 40 in the lung and oral cavity, 4 in the lung and bloodstream, 1 in the lung and urinary tract, 1 in the intestines and oral cavity, 1 in the intestines, and 1 in the urinary tract). Also, 26 (5.7%) people were infected in three sites (23 in the lung, intestines, and oral cavity, 2 in the lung, intestines, and bloodstream, and 1 in the lung, oral cavity, and bloodstream).

### 2.4. Proven and Probable IFD

Patients had proven IFD when cultures from a sterile site were positive (*N* = 8 for blood and *N* = 3 for urine). IFD was considered as probable when there were radiological signs and positive biomarker (G/GM test) or culture (nonsterile site) (*N* = 44). IFD was considered as possible when there were radiological signs without mycological evidence (negative biomarker or culture) (*N* = 114). IFD was considered as undefined when there was only clinical evidence of IFD (*N* = 289). No patient met the criteria of IFD at the start of the antifungal therapy. There were 19 patients with proven or probable IFD (7.9%) in the empirical treatment group, a significantly lower number than the 36 patients (16.4%) in the diagnostic-driven treatment group (*p* < 0.05) (see Table 4 for details).

### 2.5. Safety of Antifungal Treatment

Of the 458 patients, 79 patients had adverse reactions, 43 (18.0%) in the empirical group and 36 (16.4%) in the diagnostic-driven group, which included hepatic impairment, renal dysfunction, phantom or visual abnormalities, hypokalemia, mental symptoms, and gastrointestinal symptoms (see Table 5 for details). There was no statistical difference between the two groups. Therapy was discontinued in 9 patients, i.e., 1 patient had hepatic impairment and mental symptoms, 3 had hepatic impairment, and 4 had mental symptoms or visual abnormalities, all associated with voriconazole, and 1 patient had renal dysfunction associated with amphotericin B.

### 2.6. Risk Factors for IFD in HM Patients

Univariate analysis showed that acute leukemia (*p*=0.025), recurrence/relapse disease (*p*=0.013), neutropenia duration ≥14 d (*p*=0.006), IFD history (*p*=0.002), and diabetes (*p*=0.001) were risk factors for IFD (Table 6). Multivariate analysis suggested that recurrence/relapse disease (OR = 11.670, *p*=0.013), neutropenia duration ≥14 d (OR = 18.340, *p*=0.011), IFD history (OR = 5.270, *p*=0.021), and diabetes (OR = 3.120, *p*=0.035) were independent risk factors for IFD (Table 7).

### 2.7. Risk Stratification and Effectiveness Comparison in the Empirical Therapy and Diagnostic-Driven Therapy

The effectiveness rate was 87.9% in the empirical treatment group and 81.7% in the diagnostic-driven group, and there was no significant difference between the two groups (*p* ≥ 0.05). Based on the results of multivariate analysis, we stratified patients according to the number of risk factors. There were 183 patients with one risk factor (group-1), while 62 with two factors (group-2) and 33 with more than 3 factors (group-3). The therapy effectiveness rate in group-1, group-2, and group-3 seven days after stopping treatment was 85.2%, 75.8%, and 69.7%, respectively (*p*=0.049) (see Table 8). In group-3, the effectiveness rate was statistically significant between the empirical treatment group (85.7%) and the diagnostic-driven treatment group (41.6%) (*p*=0.0008). The effectiveness rate of the empirical and diagnostic-driven groups was 85.1% and 85.4% in group-1 and 80.6% and 70.9% in group-2, respectively. There was no significant difference in the effectiveness rate for group-1 and group-2 (*p* ≥ 0.05) (see Table 9 for details).

## 3. Discussion

Invasive fungal infections in patients with HM are a major challenge for hematologists and are a frequent cause of morbidity and mortality [13]. This retrospective study aims to compare the effectiveness of different antifungal strategies based on risk stratification.

Patients with IFD have varied underlying diseases as a function of ethnic, regional, and other differences. In this study, AML, ALL, and NHL accounted for the highest proportion, which is similar to the findings in the CAESAR [14] and SEIFEM [15] studies. The most common pathogens in IFD are *Candida*, *Aspergillus*, and *Cryptococcus* [16], with *Candida* and *Aspergillus* being the main pathogens in HM patients. *Mucor* and *Fusarium* are less frequent, but their proportion has increased in recent years [17]. In this study, the main fungal pathogens were *Candida* (93.4%), with *Candida albicans* accounting for 75.6%, followed by unclassified *Candida*, *Candida tropicalis*, *Candida glabrata*, and *Candida krusei*, with results similar to those obtained in another study from China [18]. In patients with HMs, candidiasis is often associated with presence of *Candida* in the bloodstream (candidemia) [19]. *Candida albicans* is also the most common cause of nosocomial fungal urinary tract infections. [20] In our study, eight cases were bloodstream fungal infections, of which 6 cases were due to candidiasis. All 3 cases of fungal urinary tract infection were caused by *Candida*. Fifty percent of invasive aspergillosis occurs in patients with HMs, and lung infection is the most common [21]. In our study, only 4 specimens (5.9%) were positive for *Aspergillus*, which may be attributed to the low positive rate of *Aspergillus* culture and the difficulty of taking deep tissue specimens [18]. Empirical therapy and diagnostic-driven therapy are currently the leading treatment strategies. Sun et al. [14], Yuan et al. [21], and Cordonnier et al. [22] compared the two treatment strategies and showed no significant difference in the survival rate between them. When analyzing the data in the absence of risk stratification, there was no significant difference in effectiveness and adverse events between the two treatment strategies, as observed in the abovementioned studies. This suggests that empirical therapy may not be appropriate for all patients. The number of patients with proven/probable IFD in the diagnostic-driven therapy group was significantly higher than that in the empirical therapy group, which indicates that the diagnostic-driven treatment is more targeted. Now the effectiveness of both strategies is still controversial. Generally, empirical therapy is initiated if persistent fever or recurrent fever is observed in patients. However, it is questionable to set the appearance of fever as the initiation point of antifungal therapy, since fever is not a specific symptom of IFD [23]. Moreover, application of empirical therapy may result in overtreatment or higher expense. However, because of more diagnostic technologies, it is possible to determine more precise initiating points for antifungal treatment. Therefore, diagnostic-driven therapy has become an alternative strategy that allows patients to receive antifungal treatment as early as possible. However, due to the insensitivity and nonspecificity of diagnostic tools, diagnostic-driven therapy still cannot be used as standard care. Both the Chinese [3] and IDSA [24] guidelines recommend empirical treatment strategies for high-risk patients.

In this study, duration ≥14 days, relapsed/refractory disease, IFD history, and diabetes were significantly associated with IFD in the multivariate analysis. This is consistent with most literature reports. In general, tissue fungal invasion is controlled through either neutrophil or granulomatous inflammation. Neutrophils are critical against fungus. The immunocompromise could significantly increase the prevalence of fungal diseases [25]. Neutropenia, caused by the disease itself or chemotherapy, and both duration and severity increase the infection risk. In patients with relapsed/refractory HM, as tumor load is high and drug resistance occurs, coupled with a stronger chemotherapy regimen, the inhibition of normal bone marrow hematopoietic cells is greater, which leads to more severe neutropenia, thereby increasing the risk of infection. Patients in consolidation therapy or disease remission have a lower risk of IFD based on immune function reconstitution. In our study, 101 patients had a previous fungal infection (22.1%). Most studies [3, 11], as confirmed by ours, have shown that previous fungal infection constitutes a high-risk factor for IFD, which may be related to the reactivation of latent pathogens. Diabetes has been suggested by some as a risk factor of IFD [26, 27]. Blood glucose induces a change in metabolic function, which increases the osmotic pressure of plasma, slows the circulation of monocytes and macrophages, and increases the occurrence of double infection with bacteria and fungi [28]. In addition to the above factors, there are also literature reports that AML, pulmonary dysfunction, advanced age, long-term glucocorticoid treatment, hypoproteinemia, and central venous catheterization are risk factors for IFD.

Most of the existing risk stratification models [5, 7, 11] classify every single risk factor into high-risk, intermediate-risk, and low-risk. Patients with hematologic diseases often have multiple risk factors at the same time, and the risk stratification model reported in the existing literature is difficult to distinguish the risk of IFD in patients and is seldom used in clinical practice. In this study, risk stratification was established according to the number of risk factors and verified the difference in the effectiveness of antifungal therapy in patients with different risk stratification. The antifungal efficacy of group-3 with three or more risk factors was significantly lower than that of group-1 and group-2 with no more than two risk factors. Empirical therapy was superior to diagnosed-driven therapy in patients with three or more risk factors, suggesting the importance of early antifungal therapy in high-risk patients. The risk stratification based on the number of risk factors can conveniently and effectively guide clinical antifungal therapy.

In summary, *Candida* infection was the most frequent in HMs with IFD and the lungs were the most common infection site. Acute myeloid leukemia was the main underlying disease in patients with IFD. There was no significant difference in efficacy and safety between empirical therapy and diagnostic-driven therapy. Duration ≥14 days, relapsed/refractory disease, IFD history, and diabetes were independent risk factors of IFD in HMs. The risk stratification based on the number of risk factors will be helpful to guide clinical antifungal therapy. The patients with 3 or more risk factors benefit from empirical therapy.

## Figures and Tables

**Table 1:** The criteria of IFD and antifungal treatment strategies in the Chinese guideline.

Diagnostic level	Host factors	Clinical and imaging manifestations	G/GM test	Microbiological examination	Antifungal treatment
Fever with granulocytosis	+	−	−	−	Empirical therapy
Undefined IFD	+	None or noncharacteristic changes	−/+	−	Diagnostic-driven therapy
Possible IFD	+	Characteristic changes	−	−	Diagnostic-driven therapy
Probable IFD	+	Characteristic changes	+	−	Target therapy
Proven IFD				+	Target therapy

**Table 2:** Patients' characteristics.

Baseline data		Empirical therapy group	Diagnostic-driven therapy group	*P* value
Sex	Male	143	142	0.27
	Female	96	77	

Age		49 (39, 60)	53 (39, 64)	0.10

Neutropenia duration		12.38 ± 10.17	10.35 ± 10.67	0.04

HMs	AML	131	102	0.29
	ALL	33	28	
	HAL	1	1	
	MDS	13	16	
	MM	10	22	
	NHL/HD	47	44	
	CML	1	2	
	CLL	3	4	

Disease status	Newly diagnosed	110	100	0.75
	CR	23	20	
	Relapsed/refractory	54	43	
	PR/SD	52	56	

Glucocorticoids for more than 3 weeks	Yes	47	44	0.91
	No	192	175	

IFD history	Yes	56	45	0.46
	No	183	174	

Diabetes	Yes	22	33	0.07
	No	217	186	

Pulmonary dysfunction	Yes	3	6	0.25
	No	236	213	

**Table 3:** Strains and distribution sites.

Strains	Sputum	Throat swab	Oral swab	Excrement	Urine	Blood	Perianal swab
*Candida albicans*	58	10	17	50	1		3
*Candida tropicalis*	7		1	3	1	6	
*Candida glabrata*				1			
*Candida krusei*				1			
*Candida* unclassified	7		1	13	1		
*Aspergillus*	3		1			1	
*Fusarium*						1	

**Table 4:** Diagnosis in the empirical therapy or diagnostic-driven therapy groups.

Diagnosis	Empirical therapy group	Diagnostic-driven therapy group	*P* value
Proven IFD	6	5	<0.005
Probable IFD	13	31	
Possible IFD	33	81	
Undefined IFD	187	102	

**Table 5:** Adverse reactions related to antifungal treatment in the empirical therapy and diagnostic-driven therapy groups.

Adverse reactions	Empirical therapy group	Diagnostic-driven therapy group	*P* value
Hepatic impairment	16	18	0.59
Renal dysfunction	1	0	
Visual abnormalities	11	5	
Mental symptoms	5	7	
Hypokalemia	12	10	
Gastrointestinal symptoms	3	2	

**Table 6:** Risk factors for IFD based on univariate analysis.

Factors		Proven/probable IFD	Possible/undefined IFD	*χ* ^2^	*P* value
Age	≥65	10	82	0.141	0.707
	<65	45	321		

Sex	Male	35	250	0.053	0.818
	Female	20	153		

Primary disease	Acute leukemia	43	253	5.022	0.025
	Nonacute leukemia	12	150		

Disease status	Recurrence/relapse	18	77	8.696	0.013
	Newly diagnosed	27	183		
	CR/PR/SD	10	143		

Neutropenia duration	<7 d	12	153	10.109	0.006
	≥7 d and <14 d	12	110		
	≥14 d	31	140		

IFD history	Yes	21	80	9.46	0.002
	No	34	323		

Glucocorticoids for more than 3 weeks	Yes	16	75	3.339	0.068
	No	39	328		

Diabetes	Yes	14	41	10.694	0.001
	No	41	362		

Pulmonary dysfunction	Yes	6	8	13.006	0.193
	No	49	395		

Hypoproteinemia	Yes	45	311	0.604	0.437
	No	10	92		

Deep vein catheterization	Yes	36	351	0.771	0.38
	No	9	62		

**Table 7:** Multivariate analysis of risk factors for invasive fungal infection.

Factor	OR	*P* value
Neutropenia duration ≥14 d	18.340	0.011
Recurrence/relapse disease	11.670	0.005
IFD history	5.270	0.021
Diabetes	3.120	0.035

**Table 8:** Overall effectiveness difference between the 3 groups (7 days after stopping treatment).

Groups	Effective	Ineffective/death	*P* value
Group-1 (*n* = 183)	156 (85.2%)	27 (14.%)	0.049
Group-2 (*n* = 62)	47 (75.8%)	15 (24.2%)	
Group-3 (*n* = 33）	23 (69.7%)	10 (0.3%)	

Group-1: patients with one risk factor; group-2: patients with two risk factors; group-3: patients with more than three risk factors.

**Table 9:** Efficacy of different antifungal treatment strategies in high-, intermediate-, and low-risk groups.

Groups	Empirical therapy	Diagnostic-driven therapy	*P* value
*Group-1*			
Effective	80 (85.1%)	76 (85.4%)	0.956
Ineffective/death	14 (14.9%)	13 (14.6%)	

*Group-2*			
Effective	25 (80.6%)	22 (70.9%)	0.374
Ineffective/death	6 (19.4%)	9 (29.1%)	

*Group-3*			
Effective	18 (85.7%)	5 (41.6%)	0.008
Ineffective/death	3 (14.3%)	7 (58.4%)	

Group-1: patients with one risk factor; group-2: patients with two risk factors; group-3: patients with more than three risk factors.

## Data Availability

The data used to support the findings of this study are included within the article.
